# A Non-mediational Approach to Emotions and Feelings

**DOI:** 10.3389/fpsyg.2019.00181

**Published:** 2019-02-05

**Authors:** Ricardo Pérez-Almonacid

**Affiliations:** ^1^Department of Psychology, University of Antioquia, Medellín, Colombia; ^2^Center of Studies and Research on Knowledge and Human Learning, University of Veracruz, Xalapa, Mexico

**Keywords:** emotions, feelings, non-mediational approach, referential bias, nominalist fallacy, behaviorism

## Abstract

The present analysis proposes a non-mediational approach to the study of affective phenomena. It starts off with the common recognition that “emotion” is not a technical term. Even so, researchers often treat it as if it were, confusing ordinary language with technical language. This leads to two problems: first, a referentialist bias, according to which we assume emotions to be something unapparent that one must infer and describe; and second, the nominalist fallacy, according to which we assume that emotions have causal effects on actions by the fact of naming them. I review some proposals to solve the problem, among which are some behavioral alternatives. Although these alternatives overcome many of the problems mentioned, they do not completely avoid them. I conclude that a strict non-mediational approach is possible and necessary. It supports the analytical separation of ordinary and technical language. Technical language abstracts relevant properties of ordinary language that become relevant parameters to model certain emotions, as they are referred to in ordinary language. I present some possible parameters and examples for consideration and conclude that the non-mediational approach is a plausible alternative that can stimulate research programs to find natural regularities in affective phenomena.

## The Problem

Emotions and feelings are a central topic in the history of psychology. Nonetheless, the study of emotion is not a well-demarcated field and, thus, leads to confusion. For example, Kleinginna and Kleinginna (1981) collected 92 definitions of the concept “emotion.” Likewise, Plutchik (1980) concluded that “there is no sense of the definitions moving in a certain direction with time” (p. 80). Recently, Izard (2010) acknowledged, after asking scholarly experts, that it is “difficult or impossible to conclude that emotion meets the standards of a scientific construct” (p. 368). He recommends that references to emotions in scientific literature should be specifically defined.

Some theorists (e.g., Widen and Russell, 2010; Scarantino, 2012) consider the problem to be a confusion between everyday and scientific uses of emotional words. The former are lay concepts, multivocal, with diffuse limits, and dependent on the cultural context (Russell, 1991, 2015). We use them to express ourselves and to communicate with others. When we say “the anger I felt made me hit him,” we understand well what we are talking about, but that expression is not so usual in other cultures. For example, Levy (as cited in Kassinove, 2013) reports that Tahitians have 46 different terms to talk about anger. Each use corresponds with a different property of the action. Likewise, Briggs (as cited in Kassinove, 2013) suggests that the Utku do not express anger. Linguistics, history, anthropology, history, philosophy, and, sometimes, psychology classify, describe, and define emotions with qualitative or quantitative tools. The work of Fehr and Russell (1984), and Cowen and Keltner (2017), illustrates this approach. Technical uses, for their part, delimit the field. They describe natural types and prescribe the inclusion and exclusion of cases. Their goal is to achieve consensus and guide scientific inquiry, facilitating prediction and control (Scarantino, 2012). In the field of emotions, there are no prescriptive definitions (see Izard, 2010; Widen and Russell, 2010), and, thus, there is confusion.

The main implication of omitting such distinctions between both languages is that scientists use ordinary terms as if they were technical. That is, they use terms that only make sense in everyday usage, as if they were descriptive terms for entities or natural kinds. Doing this implies at least two types of errors: (1) a referentialist bias and (2) the nominalist fallacy.

### Referentialist Bias

Referentialist bias is the assumption that ordinary words always refer to entities or activities, whether they are observable or not. Wittgenstein (1953) was especially critical of referentialism. As the noun “apple” refers to the fruit we see on the table, we could assume that the noun “emotion” refers to an entity as well. As the verb “to run” refers to the activity we see a person doing in the park, we use “feel” as if it were also an activity. We use these terms as if we had to identify and describe them in reality.

The displacement of the reference from the observable to the unobservable sustains the bias—for example, “apple” could refer to the apple on the table or to an apple in another room. We assume that the referenced unobservable items exist, but they are not accessible to our observation. Another example is having a stone in my shoe, which I refer to but cannot be observed by others. In cases like this, we rely on some other evidence, or the help of extra instruments, to confirm its existence. I call entities that are not apparent now, but could be visible later, *unobserved observables*. We can infer their functioning from direct indicators.

The usage of emotional terms exemplifies the referentialist bias when we assume they describe discrete, unobserved, but potentially observable entities. We assume that we only observe one part (i.e., physical indicators) and infer the rest because it is assumed to occur under the skin. That location is inaccessible to us, but not for the person experiencing the emotion. This person would have privileged access through a kind of internal perception. We refer to it as “to feel” or “to have the experience of,” as if it were a kind of observation that we report.

Treating emotions as if they were entities or processes happening within the skin is committing what Ryle (1949/2009) calls a “category mistake.” The example of the university illustrates the concept. A visitor comes to the campus for the first time, and the host shows him the colleges, libraries, and departments. The visitor then says that he was already acquainted with all that, but he has not yet seen where the university is. The host explains to him that the university is not a place, but a way in which those things—the colleges, libraries, and departments—are organized. The category mistake is treating the university as if it were one of the buildings. The visitor cannot see the university in the same way he sees a building—not because it is behind something, but because it is an abstract concept. Concepts are not the kind of things that we observe. One can interpret a concept, but not observe it directly—even though to interpret a concept like university one must observe things (such as buildings, boards, books, etc.). Emotions are concepts, not entities or processes occurring within an organism. The referentialist bias often implies the reification fallacy when we attribute to concepts the properties of entities, such as location, duration, and action (see Hayes and Fryling, 2017, for an akin relational non-organismic account of feelings). I now turn to the nominalist fallacy.

### Nominalist Fallacy

The nominalist fallacy consists of assuming that naming a thing also serves to explain it. For example, when someone does not get out of bed for several days and prefers to be alone, we describe it as depression. It is a label that summarizes what is happening. But we often accept it as an explanation of what we see: “He does not get up because he suffers from depression.” In general, we accept emotions and feelings as explanations of the acts that help us to identify them. It is circular reasoning because the evidence for depression is the behavior we observe (Schlinger, 2013).

We commit the nominalist fallacy and the referentialist bias when we refer to emotion as the cause of action. This is often the case in folk psychology (Ong et al., 2016). We accept it when we wish to understand a situation, because we usually accept reasons as explanations. But we cannot demand that lay people conform to the logical demands of scientific language. Instead, if we want to explain depression, we ask why people do not get out of bed and what led them to that condition. We discover causal explanations when the answer points to something other than the depression itself.

### Some Examples

Several examples show that it is common for scientists in the so-called affective science to fall into the problems described above. The following cases illustrate these tendencies.

In Finucane’s study (2011), researchers presented a scene from a movie that caused anger. Then, they presented images from the scene at regular intervals during a task to measure attention. The participants evaluated how much anger they felt. The researchers verified that the participants rated higher levels of anger in front of the anger-inducing scene, and that the reaction times in the task were faster than in the control condition. Those results would show enhanced focusing of attention. The author concluded: “These results support the general prediction that high arousal negative emotional states inhibit processing of non-target information and enhance selective attention” (p. 973). Thus, anger is an unobserved state that we recognize through words and actions. It is something inferred beyond the measured behavior, which exemplifies the referentialist bias. However, we expect in science that entities have clear limits, in order to achieve replicable generalizations. But which of the 46 forms of anger described by the Tahitians correspond to the generalization reached by Finucane (2011)? Does the generalization not apply to the Utku, who, according to anthropological evidence, do not express anger?

On the other hand, attributing causal effects (inhibit and enhance) to the concept of “anger” on a property of the action (selective attention) illustrates the nominalist fallacy. If the author does not specify anger as an entity or activity distinct from the action that serves to identify it, then anger could not be the cause of the action. Indeed, if we wish to replicate the effect on selective attention, then we would manipulate the film, not the emotional state. Thus, what is relevant for scientific explanation is to describe how some parameters of the film affect the performance in the task.

Polman and Kim (2013) provide another example. They asked their participants to describe something they had done the previous day, and then report whether their current emotions and feelings were angry, disgusted, sad, or neutral, on a scale of 1–5. Finally, they responded to a social dilemma in which they could keep chips, give them to a group pool, or take them from the pool. Depending on whether there was an excess or deficit of chips in the pool compared with the number taken, they received a bonus or not. The authors measured the effect of certain emotions on the amount of chips participants gave or took. They found that the participants who had reported sadness donated more resources to the group, but also took more from it than the participants who reported an emotionally neutral state. The authors concluded that: “This indicates that sadness has mixed effects on decisions to cooperate in a social dilemma. Sadness causes individuals to give more shared resources to others, but also take more shared resources for themselves” (pp. 1688–1689).

Unlike in the study by Finucane (2011), in Polman and Kim (2013), emotion was not induced by a manipulated external event. They began their analysis from the participant’s report. However, the logic of the analysis was the same: sadness is a discrete phenomenon different from adding chips to, or removing chips from, the group pool. We do not have access to the sadness except through the participants’ reports. Intuitively, we agree that what we call sadness is something, and therein lies the referentialist bias. We encounter the nominalist fallacy when authors explain giving or taking resources by naming and appealing to sadness. The only evidence we have that sadness is something is that we treat it as one in our linguistic practices. But practices are variable and circumstantial. For example, in some African languages, there is only one word that covers what in English is both “anger” and “sadness” (see Bamberg, 1997). In conclusion, affirming that sadness causes action is an expression that makes complete sense in ordinary language, and we can analyze it as such. But it would not be the kind of conclusion one hopes to get from a scientific study of emotions.

## Some Alternative Solutions

The solution to this problem lies in studying emotions without treating ordinary language as if it were technical. Several authors have proposed different solutions, and I will review some of them below. The behavioral alternative will be analyzed in more detail because it is the direct antecedent of the non-mediational approach suggested later. The success of the proposed solutions depends on how much they avoid the two errors discussed in the previous section. The thesis is that none of the proposed solutions eliminates either error completely, and that we need a strict non-mediational approach instead.

### Scarantino’s Scientific Emotion Project

Scarantino (2012) distinguishes between the Folk Emotion Project and the Scientific Emotion Project. The former describes the membership conditions of ordinary emotion categories. The latter prescribes membership conditions of natural kinds of emotions. A natural kind is a category containing the maximum class of items that share properties for a scientific explanation by causal mechanisms. Most members of a natural kind are also part of the ordinary categories, so the relationship between the two is one of *subordination*. For example, the categories “anger_x_” and “anger_y_” are different natural kinds of the ordinary category “anger.” So most of the members of the first category are also members of the second one. One issue is that most members of a natural category also belong to an ordinary category. But if the limits of the latter are fuzzy, culturally dependent, and do not refer to a given entity, then the limits of the former are also quite undefined. It goes against the aspiration that they serve as a strict scientific language.

### Russell’s Constructionist Perspective

Russell (2015) proposes a unit of analysis called “emotional episode,” which is not qualitatively different from a non-emotional episode. Thus, it is not a technical term but a class of phenomena ordinarily defined. Psychology explains the emotional episode using the same technical language that would be used to study any other kind of episode. An emotional episode is a one-time event, constructed out of simpler components. A component is whatever an observer takes as a sign of emotion—for example, core affect, emotional meta-experience, affective quality, appraisal, or instrumental action. Therefore, the relationship between ordinary and scientific language is one of *application*. The former defines the phenomenon under investigation, and scientists apply the latter to understand it. At first glance, one problem is the *a priori* assumption that an “emotional episode” includes concepts like core affect and appraisal, which carry an important theoretical load. The referentialist bias lies in the supposed components of an episode ordinarily conceived.

### Behavioral Approaches

Behavioral approaches consider emotions as properties of behavior, rather than as another kind of phenomena. The function of technical-scientific language is to describe behavior. Thus, behavioral scientists translate any ordinary reference to emotions into that behavioral, technical language. The relationship between both languages, then, is one of *translation*.

Duffy’s (1941) classic ideas are compatible with this approach. She suggests that “emotion” is not a natural category. Instead, the term designates extreme values of the vigor of responses in situations interfering with the achievement of a goal. These ideas did not lead to an experimental program, so it is difficult to check its heuristic scope. However, the Skinnerian behavioral tradition offers us enough material for analysis due to its vast theoretical and experimental production.

Skinner laid his groundwork for the relationship between ordinary and technical language in a seminal paper (1945), which he further developed in *Verbal Behavior* (1957), and revised in his last years (Skinner, 1984). His alternative was the functional analysis of ordinary psychological terms: dealing with ordinary terms as verbal responses, and finding out the conditions under which people utter them and the verbal community reinforces them. Therefore, through functional analysis, we translate ordinary terms into the scientific language of the occasion-response-consequence schema. This was his alternative to classic operationism, which “[has not] improved upon the mixture of logical and popular terms usually encountered in casual or even supposedly technical discussions of scientific method” (Skinner, 1945, p. 270).

In particular, Skinner (1953) proposed translating ordinary emotional terms as predispositions to act in certain ways. For example, saying that a man is “angry” can mean that he is more likely to hit or insult someone in certain conditions. Translation is not one-to-one: “Even an apparently well-marked emotion like anger may not be reducible to a single class of responses or attributable to a single set of operations” (Skinner, 1953, p.164).

The functional analysis of ordinary terms surpasses both the referentialist bias and the nominalist fallacy. Apropos of the first, Skinner (1953) affirmed: “The names of the so-called emotions serve to classify behavior with respect to various circumstances which affect its probability... by describing behavior as fearful, affectionate, timid, and so on, we are not led to look for *things* called emotions” (p. 162). As regards the second, the author stated:

A man does not neglect his business because of anxiety or worry. Such a stamen is at best just a way of classifying a particular kind of neglect. The only valid cause is the external condition of which the behavior of neglect, as part of an emotional pattern known as anxiety or worry, can be shown to be a function (p. 168).

Thereby, functional analysis has hitherto been a reliable alternative to overcome these logical problems. However, we need another non-mediational approach to the study of emotions for two reasons: (1) operant functional analysis alone seems to be insufficient and (2) certain modalities of the referentialist bias persist in this approach, although not in a reificationist mode. Specifically, sometimes behavioral scientists have treated emotions as states that operate concurrently with behavior, or as affective experiences.

### Emotions as States in Functional Relationships

At times, Skinner included emotions as part of a functional relationship: “We discover the variables of which emotional states are a function—as we discover any variables—by looking for them [...] Continued physical restraint or other interference with behavior may generate ‘rage”’ (Skinner, 1953, p. 164). Consequently, the emotional states are no longer ordinary translatable terms. Instead, Skinner uses “emotion” as a concept that is part of the scientific scheme of behavior. It is an event that is a function of something, or can be generated by something else.

Skinner (1974) also says that “[behavior] does not change because [the individual] feels anxious; it changes because of the aversive contingencies which generate the condition felt as anxiety. The change in feeling and the change in behavior have a common cause” (Skinner, 1974, pp. 61–62). It is clear that Skinner does not consider the feeling of anxiety to be the cause of the behavioral change. Instead, both are co-occurring products of the same contingency. However, it is also evident that both are two different things: one is a felt condition, and the other is overt behavior.

One more example of this usage is evident in the classic study by Estes and Skinner (1941). They evaluated the effect of anxiety on the steady response rate in a positive reinforcement schedule, and defined anxiety as an anticipatory emotional state. A Pavlovian tone-shock pairing procedure served to establish the conditioning of a state of anxiety. The effect found was a decrease in the response rate. The authors explained: “Anxiety is an emotional state arising in response to some current stimulus which has been followed by a disturbing stimulus. The magnitude of the state is measured by its effect upon the strength of hunger-motivated behavior” (p. 400).

### Affective Experiences

Skinner (1953, 1957) addressed what in ordinary language we call affective experiences. He showed us how to translate them into the scientific schema of behavioral science. The distinction between public and private events served this purpose. The criteria of distinction between both were location and accessibility. If events occur within the skin, they are private and thus are difficult to access; if they occur externally, they are public and accessible. Of course, he took pains to make it clear that the same laws governed private and public events, and ended up yielding to the idea that something happens under our skin, and we have to account for how it comes under the control of contingencies of reinforcement (see a critical analysis in Hayes and Fryling, 2009).

Skinner (1957) treated both unobserved observables and concepts as private events. For example, he states:

If we could say precisely what events within the organism control the response *I am depressed*, and especially if we could produce these events at will, we could achieve the degree of prediction and control characteristic of verbal responses to external stimuli (p. 131).

Skinner considered that an event within the organism controls the expression “I am depressed,” as a stone controls the expression “I have a stone in my shoe.” The person describes something happening under the skin, which we are accustomed to refer to as “depression.” This creates a category mistake by confusing a concept with an unobserved observable.

Perhaps Skinner did not intend to do a philosophical treatment of privacy. Instead, he wanted to show that we can functionally analyze the way we learn to talk about private events. However, the logical consequences of his approach are remarkable, even today. Privacy remains a matter of controversy among behavioral analysts (Schlinger, 2011). The way the study of emotions and feelings has evolved suggests that the approach has not changed substantially. For example, Baum (2011) analyzed the inaccuracy of introspection and stated: “People often express confusion or uncertainty about private events (Is that a pain or an itch? Am I embarrassed or angry?)” (p. 190). Thus, Baum exemplifies private events (events occurring within the skin, p. 186) with concepts like embarrassment and anger, tacitly suggesting that they are something we could refer to, and that the verbal community infers (p. 190).

Several contemporary behavior analysts ratify the referentialist bias when studying emotions and feelings. They place feelings within the field of experience, as behavior controlled by private stimuli. In doing so, they mix the categories of ordinary language categories with those of the operant conditioning. For example, Layng (2017) distinguishes between emotions as private experiences and overt behavior. Likewise, Moore (2000) explains the relationship between private experiences and behavior. According to him, some contingencies result both in a change to the probability of responses, and the feeling of bodily conditions (note here the connection to W. James’ theory—James, 1884). What is “feeling” here, apart from behavior? Whatever it is, what the person feels acquires a discriminative function. The verbal community reinforces certain expressions when the person is feeling. That expression then acquires discriminative functions for further behavior, and so on. So, feelings (an ordinary category) become intertwined with behavior (a scientific category). Authors such as Friman et al. (1998) present an even more complex picture. They deal with private experience as a consequent event: “Consistent with our experiential avoidance approach to anxiety disorders, the disruptive washing has important functional properties: reducing, avoiding, or escaping private events (experience)” (p. 148).

Keller and Schoenfeld (1950) approached emotions as behavioral changes resulting from certain operations—for example, interrupting a behavioral chain. Authors such as Dougher and Hackbert (2000) and Lewon and Hayes (2014) have developed this idea further. They use the concept of establishing operations (Michael, 1993) and that of motivating operations (MOs) defined by Laraway et al. (2003). This approach coincides with a non-mediational one in its parametric emphasis. Even so, it still falls into a referentialist bias because it includes feelings and emotions as something operating in contingencies of reinforcement. For example, Lewon and Hayes (2014) consider that events that serve as MOs “are often correlated with the report of subjective feelings we learn to describe emotions or moods [...] the same events that are said to produce the subjective feelings of emotions or moods also serve as MOs....” (p. 817).

Finally, other behavioral traditions fall into a referentialist bias when they translate emotions as behaviors or properties of behaviors. That bias is not “inward,” as a reference to an unobservable private event, but “outward,” as a reference to a public event. As a consequence, they understand emotions as emotional behavior—which is different from a supposed non-emotional behavior—or as properties of responses specified episodically. This approach is especially noticeable in some classical authors, such as Watson (1914, p. 185), and Kantor (1933, p. 201).

## A Non-Mediational Approach to Emotions and Feelings

The present analysis is akin to other non-mediational approaches, such as Watkins’ (1996) for memory and Gibson’s (1979) for perception. Watkins (1996) shows that scientists use “memory trace,” which is a metaphor, as a hypothetical construct causing remembrances. This construct illustrates both the referentialist bias and the nominalist fallacy. The former by assuming that it is something simply because we refer to it; the latter by suggesting that we are explaining remembering when we name it in a causal relation. The result is an inexhaustible source of theories with no critical test to discriminate among them. Watkins’ alternative is to discard concepts of this sort and instead proposes “... identifying, weighing, and deciphering the relation between the key variables operating at the time of an event’s occurrence and at the time of its recollection” (p. 331).

For his part, Gibson (1979) remarks, critically, that the traditional psychology deals with the concept of “sensation” as a descriptor of something for which we must account. This information would be the data on which a process ending in perception operates. The result is the explosion of sophisticated theories to explain how we perceive from these supposed sensations. Thus, a concept that makes sense within social practices enters into causal relations as if it were an empirical entity. Gibson’s alternative involves a richer conception of the environment and the perceptual systems, and the description of the direct relationship between both.

Mediationalism posits that perception results from operating on visual sensations, while remembering is the outcome of processing on memory traces. In contrast, non-mediationalism considers perception to be the direct relationship with places, objects, and events currently occurring, while remembering is the direct relationship with events which have already occurred. This alternative focuses on describing that relationship, and we must specify the relevant environmental parameters. The relationship involves not “stimuli” but, rather, more complex properties of environmental events such as persistence and change, gradients, and the proportion of classes of cues. Despite the epistemological closeness with behaviorism, Gibson and Watkins consider it too narrow to cover such matters sufficiently.

Regarding the relationship between ordinary and technical language, the approach proposed here holds that it is one of *abstraction*: technical language abstracts specific properties in ordinary language (cf. Ribes, 2018). I suggest that this way of conceiving that relationship would reduce the risk of falling into the problems mentioned. It will be further developed in the following sections.

### The Grammar of Ordinary Expressions

Ordinary language is as good as it could be because it has evolved to communicate meaningfully, not to do science. “I feel angry with him,” “I experienced a deep frustration with the project,” and “She has been feeling depressed” are expressions that make sense. Ryle (1949/2009) and Wittgenstein (1953) give us some tools for analyzing those expressions. The result is to recognize in them some properties relevant to scientific study.

Based on the ideas of Wittgenstein (1953) and Ter Hark (1990), it is possible to state that words are different concepts if their usage is also different (see also Slaney and Racine, 2011). A *language game* (Wittgenstein, 1953) or field of application defines a word’s use. When we learn to talk about emotions and feelings, we learn concepts (not definitions), just as we learn concepts such as *poverty*, *justice*, *university*, *belief*, and so on. Learning to talk about emotions is no different from learning to talk about other concepts. Thus, “I am depressed,” which Skinner (1957) used as an example of a reference to private events, seems to be more of an abstract tact than a reference to a private event. The reason is that it expresses subtle properties of a social practice and not an event under the skin.

The relationships between concepts, expressions, and actions in a language game are internal relations to such a game. Ter Hark (1990) defines internal relationships by three characteristics. First, it is impossible for the two relata not to have such a relation. The reason is that the criterion for the identity of one is the criterion for the identity of the other. When we talk about emotions, it is not possible to identify an emotion without referring to some degree of action. It is also not possible to identify an action without some degree of emotion (e.g., “kiss with passion,” “write with frustration,” and so on). Second, a third term does not mediate the relationship between the two relata. The relation is direct. That is, it does not need to include a term that makes an adverbial relation possible. By doing that, it externalizes the internal. This would create hypothetical constructs leading to a regression to infinity. Finally, the relation exists within a practice. A social practice establishes the relation between emotion and action. It is not something generic, because both are concepts coined in particular cultures in a certain way. So the internal relations of a language game constitute the grammar of ordinary expressions.

The description of the grammar of ordinary emotional expressions aims to understand, not to explain (von Wright, 1971). Thus, art, anthropology, philosophy, and linguistics can do it successfully. Statistical classification of ordinary emotional concepts (e.g., Cowen and Keltner, 2017) is also an example of this type of inquiry.

### Natural Regularities

External relations have the three characteristics opposed to those of internal relations. That is: (1) the relata are independent, and their relation is contingent; (2) a third element could mediate the relation; and (3) the relation exists beyond a particular social practice. Natural sciences aim to find external relations, and causality is a type of them (von Wright, 1971). We commit both the referentialist bias and the nominalist fallacy when we include a concept within an external relationship.

Now I can revisit the studies that illustrate the problems analyzed above. In our terms, Finucane (2011) found that a person who is angry performs the attentional task in a certain way. Performing in that way is a property of anger, not an effect of it. Based on Finucane’s study, we know that solving that task in that manner could be a criterion to interpret that a person is angry. However, we still do not know how or what is the cause. To know that, we need to look at something external to that relationship—in this case, the film. Proposing a hypothetical extra mechanism does not solve the problem but complicates it. Though this analysis could be extended to Polman and Kim’s (2013) study, they do not offer enough information about possible external relations.

### The Relationship Between Ordinary Language and Scientific Language

The relationship between ordinary and scientific language is one of *abstraction* (Ribes, 1991, 2018). We abstract some properties in the internal conceptual relations entailed in the expressions of ordinary language through the lens of the scientific language. Those properties will allow us to find orderly external causal relations. The result is that we achieve an *experimental model* of emotion, in whatever form ordinary language expresses it. Ordinary language provides the phenomena that attract the interest of psychology. Scientific language offers concepts to isolate relevant properties in order to find scientific regularities. Thus, the latter must include concepts referring to independent entities. Otherwise, they could not be part of external relations, and the logical problems already analyzed would persist.

### The Basic Analytical Unit

The unit of analysis is an organism and an object or event with which it is in a relationship. Therein, I agree with the behavioral tradition. It is not necessary to the analysis that such a relationship is operant behavior, although the analysis could include it. Some authors, such as Roth and Gewirtz (1998) and Layng (2017), defend the need for research on emotion as operant behavior. Such research focuses on studying the roles of discriminative stimuli, responses, consequences, and establishing operations. The alternative considers that this analytical unit does not frame all the relevant problems. As Kantor (1970) points out:

We may not regard conditioning or any other single kind as the necessary and sufficient way to deal with all behavior. To do so means uniformly to reduce all behavior to a single class adaptable to arbitrary chosen patterns of manipulation and specialized apparatus (p. 102).

For example, to develop an experimental model of Schadenfreude (joy in others’ misfortune, Smith, 2013), response rate may be insufficient or even irrelevant. Skinner (1950) argued that it is the best alternative to study learning, but other phenomena may need other types of measures.

An organism relating with an object or event as the unit of analysis is common to the study of any psychological phenomenon. The particularity in the analysis of emotions is what we observe in such a relationship.

### The Relevant Parameters to Study Emotions

The properties abstracted from the ordinary language are considered parameters of the individual and the events. Parameters are qualitative and quantitative dimensions modulating the relationship between individuals and events.

The individual’s parameters are any property of their reactivity. We can measure them as magnitude, duration, persistence, amplitude, variation, effectiveness, precision, latency, organization, and direction, among others (Ribes, 2018). A single parameter can hardly suffice for an experimental model of emotions. Authors like Sherman (as quoted in Keller and Schoenfeld, 1950 and Russell, 2015) report that we cannot identify a particular emotion based on one reactive pattern. Thus, it is helpful to return to Kuo’s (1967) concept of *behavioral gradients*. This refers to the qualitative and quantitative variations of the different reactive systems involved in the behavior of an organism. In some cases, certain systems are dominant, and in others, other systems will be dominant. However, there is always activity in all the reactive systems (Kantor, 1933).

On the other hand, choosing the parameters of the relevant event is critical to developing an experimental model of emotions. This is the crux of the matter. For example, Keller and Schoenfeld (1950) think that removal of reinforcers is relevant to the study of anger. We could refer to them as MOs (Laraway et al., 2003) or as setting factors (Kantor, 1933), if this facilitates the analysis. However, rather than thinking about a list of operations, the critical issue is to abstract parameters related to affective expressions in ordinary language.

Roseman (2001) proposed parameters that he considered appraisal dimensions of emotions. Using them as such entails hypothetical causal processes and thus falls into the nominalist fallacy. Instead, we can consider those “dimensions” as situational parameters. For his part, Turkia (2009) offers other parameters in context of the computational modeling of emotions and feelings. Turkia’s epistemological assumptions do not match those of the present proposal either. However, the author thought of those parameters as algorithms, which makes it easier to incorporate in the present analysis. Thus, I offer an adapted and completed list of event parameters that could be useful to the development of experimental models of emotions and feelings. Some of them were borrowed from Roseman (2001) and Turkia (2009):

(1) *The nature of the event:* It is that with which the individual interacts; it can be an event or an object.
(2) *Direction:* If it is an event, whether it is directed toward oneself or others.
(3) *Intensity:* Magnitude of occurrence.
(4) *Time:* If it is an event, the moment of its occurrence: whether it already happened, is currently happening, or will happen.
(5) *Probability:* The degree of certainty (certain or uncertain) that the individual has about the future occurrence of the event.
(6) *Predictability:* The extent to which the event belongs to a pattern or regularity. It can be high or low predictable.
(7) *Delay:* Time elapsed since occurrence and affectation.
(8) *Agency:* What or who produced the event. It can be produced by circumstances, other individuals, or oneself.
(9) *Potential control:* Whether or not the individual can alter the occurrence of the event and its properties. Potential control could be high or low.
(10) *Motivational condition:* Whether the individual would continue interacting with the event or object or not.
(11) *Motivational compatibility:* Whether an event or object is compatible with the motivational condition. It can be consistent, inconsistent or irrelevant. If it is inconsistent, then the problem type needs to be specified (see below).
(12) *Problem type:* Whether inconsistency arises because an event blocks the completion of an interaction, or by some other variable intrinsic to the event.

### Some Examples

We could model some ordinary episodes expressed as hope, desire, or impatience. For instance, consider a situation in which an individual interacts with an event, which has the following properties (parameters):

(1) It is highly predictable.
(2) Its probability of occurrence is uncertain.
(3) It is consistent with a condition relevant to the individual at that moment.
(4) If established, it would lead to the individual remain in the situation.
(5) It is caused by the circumstance or by another individual.
(6) It is not under the control of the individual.

If we vary the probability of occurrence, ceteris paribus, we would map other ordinary situations usually expressed, such as joy or delight.

On the other hand, ordinary episodes that could be called “disappointment” could entail a situation in which an event has already occurred, and it was directed toward the individual, and was predictably consistent with a motivational condition at a lesser magnitude than predicted. A similar emotion in ordinary language, such as “bewilderment,” may require changing the parameters for its experimental modeling. In this case, we would design a situation in which a highly predictable event stops occurring or occurs in a different way than usual for circumstantial reasons or because other individuals, it was directed at the individual, and the individual had little control of its occurrence, regardless of its motivational compatibility.

In Estes and Skinner’s study (1941), instead of anxiety affecting the strength of behavior, we can design the situation parametrically: an event highly regular, directed to the rat, motivationally inconsistent, whose probability is uncertain, caused by the circumstance, and with a very low potential control. Possibly, that the decrease in the response rate depends on the degree of predictability of the event—which we could manipulate, the uncertainty of its occurrence, or the degree of potential control over its occurrence.

We can model expressions referred to emotions, passions, feelings, moods, and so on, combining some parameters. As Ryle (1949/2009) noted, in ordinary language we refer to different types of things when talking about emotions—we mean states, dispositions, occurrences, or relationships. We can model them through infinite measures and manipulations. For example, when someone causes an event that blocks the achievement of a highly preferred condition, and we measure the magnitude, duration, direction, persistence, and organization of the activity, this could be a model of “feeling anger” as a sudden and momentary state. The temporal evolution of those measurements, while we hold the parameters constant, or increase them, could correlate with a range of denominations of anger. If we then arrange the situation so the individual continues the interrupted task and we continue measuring those behavior properties, a different pattern might emerge. It is no longer a temporary state but a disposition to do things in a certain manner. It would no longer be an emotion in ordinary language, but a “feeling of determination,” for example.

Finally, the systematic analysis of the behavioral gradient development raises a number of productive questions: how are different components of the same reactive pattern differentially affected among themselves? How does the induction of certain responses (facial expressions, postures, gaze, and so on) mobilize a complete pattern in which they are already integrated (see Laird, 2007)? How can certain instrumental skills alter the parameters of the situation modulating certain properties of the behavioral gradient? The answers to all of these questions could also have enormous clinical potential.

## Conclusion

It seems that a strict non-mediational analysis of emotions and feelings is possible. Its core is the search for functional relationships between parameters, i.e., conceptual properties abstracted from ordinary emotional expressions. Using this approach eliminates the most common problems in the experimental analysis of emotions. This analysis’ aim is the explanation of natural phenomena, since it does not introduce ordinary concepts as part of functional relationships. However, this approach recognizes the usefulness of the interpretative analysis of such ordinary concepts for understanding how we talk about emotions and feelings, but not for explaining them. Finally, we could formulate law-like regularities, which are still absent in the literature on emotions and feelings.

## Author Contributions

RP-A conceived and developed entirely the ideas exposed in the document.

## Conflict of Interest Statement

The author declares that the research was conducted in the absence of any commercial or financial relationships that could be construed as a potential conflict of interest.
